# Editorial: A granular approach to internalizing disorders in adolescents: Examining developmental pathways and environmental influences to identify high‐risk youth

**DOI:** 10.1002/jcv2.70000

**Published:** 2025-03-02

**Authors:** Pablo Vidal‐Ribas

**Affiliations:** ^1^ Child and Adolescent Mental Health Research Group Institut de Recerca Sant Joan de Déu (IRSJD) Barcelona Spain

**Keywords:** adolescence, anxiety, depression, females, internalizing, risk

## Abstract

Adolescence is a critical developmental period during which internalizing disorders, such as depression and anxiety, frequently emerge. This editorial highlights six studies from this issue that investigate the complex interplay of genetic, physiological, cognitive, social, and environmental factors contributing to these disorders. By employing robust methods, these studies inform on mechanisms and trajectories, enhancing the identification of high‐risk individuals for targeted interventions. Together, these studies emphasize the importance of nuanced, multi‐factorial approaches in advancing research and practice in adolescent internalizing psychopathology. Nevertheless, future research must prioritize diverse samples, dynamic longitudinal designs, and multi‐informant approaches to increase generalizability and impact.

**Key points**

- Adolescent internalizing disorders arise from genetic, physiological, cognitive, social, and environmental factors, requiring comprehensive research approaches.
- Detecting high‐risk individuals early allows for timely, targeted interventions to prevent worsening mental health outcomes.
- Mediation, moderation, and trajectory analyses help uncover risk factors, developmental pathways, and symptom variations, improving early detection.
- Overreliance on self‐reports, limited multi‐informant data, and a lack of diverse study populations reduce the accuracy and generalizability of findings.
- More diverse samples, longitudinal designs, and multi‐informant approaches are essential to refining risk assessment and ensuring equitable mental health support.

## INTRODUCTION

Adolescence is a critical developmental period marked by major hormonal, physical, neural, cognitive, and social changes. It is during this time, from the early teens to the mid‐twenties, that many mental health problems first emerge; studies estimate that nearly 50% of people with mental health problems experience their first disorder before the age of 18, with the peak onset occurring around 14.5 years of age (Solmi et al., 2022). The increase in mental health problems during adolescence is particularly significant for internalizing disorders, an umbrella term traditionally encompassing mood and anxiety/stress‐related disorders. Recent nosology frameworks such as HiTOP have expanded this definition to include sexual problems, eating disorders, and suicidal thoughts and behaviors.

Despite this overarching construct, there is considerable variation in the types of internalizing disorders that emerge during adolescence, the mechanisms underlying them, and the individuals affected. It is therefore imperative for studies to consider such heterogeneity to identify early and accurately those children that will develop internalizing psychopathology in adolescence. The six original articles featured in this issue of *JCPP Advances* (March 2025) tackle this challenge by examining the interplay of individual vulnerabilities—including biological, affective, and cognitive factors—alongside social and environmental influences. Through robust statistical methods and fine‐grained approaches, these studies offer new insights to enhance early identification and targeted interventions for children and families who need them most.

## SUMMARY AND DISCUSSION OF ARTICLES IN THE CURRENT ISSUE

One way to identify high‐risk individuals in developmental psychopathology is by examining the influence of third variables, mostly identified through mediation and moderation analyses. While these methods are often confused, they examine different aspects of a relationship. Mediation explores whether an intermediate variable explains the relationship between an independent variable (e.g., individual vulnerability) and a dependent variable (e.g., internalizing psychopathology), providing insight into *how* or *why* an effect occurs (i.e., the mechanisms). In contrast, moderation investigates whether the strength or direction of this relationship changes depending on the level of a third variable, revealing *when* or *for whom* an effect occurs (i.e., the conditions).

For example, in a preregistered study, Chiu et al. (2024) used mediation analysis in a UK sample of 2397 adolescents to show that depressive symptoms at the 1‐year follow‐up mediated the relationship between baseline social anxiety and depressive symptoms or suicidal ideation 2 years later. Similarly, Harrewijn et al. (2024) used data from the Generation R Study to show that parent‐reported (but not observed) fearful temperament was associated with anxiety symptoms in adolescence, and this association was moderated by negative feelings after a social exclusion lab‐based task, but not by sex, emotional responses during the task and other peer interaction variables in childhood. The findings from these studies highlight the value of identifying mediators and moderators to identify high‐risk individuals and interventions targets, such as socially anxious youth with depressive symptoms or children with early fearful temperaments whose distress from social exclusion lingers longer than expected.

One of the biggest challenges in studying developmental psychopathology is the variability of mental health problems during the first two decades of life, both between and within individuals. This variability, though, opens the door for the identification of individuals who are at higher risk of developing symptoms or even those that are more resilient over time, who can inform prevention efforts. To do so, researchers often use data‐driven methods like Growth Mixture Modeling to analyze symptom trajectories. For example, Ravagnani Salto et al. (2024) studied anxiety trajectories in the Brazilian High‐Risk Cohort for Mental Conditions (mean age at baseline, 10.4 years) over 9 years, identifying three patterns: high‐decreasing (7.6%), moderate/low‐stable (89.6%), and low‐increasing (2.9%). Lower IQ predicted high‐decreasing anxiety, while higher IQ and female sex were linked to low‐increasing symptoms. Of note, polygenic risk scores (PRS) for anxiety, depression, and well‐being showed no association with trajectories. Similarly, Donaldson et al. (2024) examined mental health trajectories during the primary‐to‐secondary school transition in a large UK cohort, finding four classes: “persistently elevated” (9%), “hyperactivity and conduct problems” (23%), “emotional and peer problems” (15%), and “persistently low” (54%). The “emotional and peer problems” group was associated with being female, recent negative life events, and transition‐related worries, while the other high‐risk groups were linked to being male, low socioeconomic status, and special educational needs. Trajectory analyses like these also help us to identify at‐risk groups for targeted interventions, such as females with high IQ, recent negative life events and worried about starting high school, who may benefit from early emotional support.

While prospective longitudinal studies are better suited to identifying precursors of mental health problems, cross‐sectional studies can highlight intervention targets and generate new hypotheses. For instance, using data from over 11,000 14‐year‐olds in the UK Millennium Cohort Study, Tsomokos et al. (2024) found that an evening chronotype (i.e., going to sleep late) was significantly linked to higher depressive symptoms, particularly in females, even after adjusting for sleep duration and other key factors. These findings suggest that improving sleep habits in adolescence could help reduce depressive symptoms. Similarly, Grasser et al. (2024) examined 48 treatment‐seeking youth aged 9–19 and found that cumulative exposure to threat‐related trauma, such as abuse, was associated with increased irritability, while deprivation‐related trauma, like neglect, did not. These results highlight the importance of incorporating threat‐related trauma in theories and treatments of irritability.

## GENERAL DISCUSSION AND FUTURE DIRECTIONS

Internalizing disorders in adolescence are complex and arise from multiple interacting factors that are difficult to comprehend when examined in isolation. The articles discussed here collectively examined genetic, physiological, cognitive, social, behavioral, environmental, and clinical factors of anxiety, depression, irritability, and suicidal ideation. Although there are many other aspects implicated and not covered by these studies, such as puberty changes, neural factors, family relationships, and gene‐environment interactions, to name a few, most of these studies used multi‐factorial approaches thus providing a more comprehensive understanding of risk. For example, Ravagnani Salto et al. (2024) integrated genetic vulnerability, intelligence, sex, maternal mental health, and exposure to threat to study anxiety trajectories. Moving forward, longitudinal studies should investigate how these factors evolve and interact dynamically over time, rather than treating them as static, to deeper insights into the best timing for intervention.

The increased risk of depression and worry‐related anxiety in females is well documented, doubling that of males by mid‐adolescence, and persisting across most adulthood (Yang et al., 2024). Nevertheless, relying solely on sex as a screening tool for internalizing disorders would obviously yield a high number of false positives, emphasizing the need of multi‐factorial approaches to refine risk assessment (i.e., which females are at higher risk?) the same way that it has been done to understand sex differences (Serio et al., 2022). Five studies discussed in this editorial examined the role of sex. All found being female to be a risk factor of internalizing symptoms, yet sex did not modify the risk carried by other factors in all cases. For example, whereas Tsomokos et al. (2024) showed that evening chronotype effects in depressive symptoms were stronger for females, Harrewijn et al. (2024) did not find sex to moderate the association between early childhood fearful temperament and adolescent anxiety. This supports again the importance of nuanced, multi‐dimensional frameworks to the study of internalizing psychopathology.

All the studies discussed here relied on self‐reports to assess internalizing symptoms, with four using them as the sole source of information. While self‐reports may be influenced by social desirability or emotional states, they remain essential for understanding adolescent psychopathology. These assessments align with the recommendation of the Lancet Commission on Adolescent Health and Wellbeing to give adolescents a voice in identifying their health issues, especially during a period when internalizing behaviors increase and parents may be less aware of their child's emotional state. That is not to say that these should not be complemented with parent‐ or teacher‐reports. Indeed, informant discrepancies can provide valuable insights. For instance, Harrewijn et al. (2024) found that parent‐reported peer interaction variables did not predict self‐reported anxiety, while Grasser et al. (2024) observed that females self‐reported more irritability than males, with no differences in parent reports. Indeed, collecting self‐ and parent‐reports might also be relevant for studying sex differences in internalizing symptoms. Data from the UK Millennium Cohort Study show that parents often overreport emotional problems in males but underreport them in females, with females showing a larger informant discrepancy (Patalay & Fitzsimons, 2017). Research highlights a disparity in parents' awareness of emotional problems in adolescent females (Sourander et al., 1999), underscoring the need for multi‐informant approaches.

Finally, diversity in study populations remains a significant gap. Only two of the studies discussed here included underrepresented communities, with just one of them being conducted in a middle‐income country. This limits the generalizability of findings and may influence the applicability of tools like PRS, as potentially demonstrates the study from Ravagnani Salto et al. (2024), which are often derived from samples with European ancestry. In addition, the expression and self‐regulation of emotions and internalizing psychopathology is impacted by cultural factors which can influence rates and informant discrepancies of internalizing disorders and left youth untreated (Kim et al., 2016). Future studies must prioritize diverse populations to ensure relevance across cultural contexts and address disparities in the understanding and treatment of internalizing disorders.

## CONCLUSION

The six studies featured in this issue of *JCPP Advances* highlight the complexity and heterogeneity of adolescent internalizing disorders, offering valuable insights into their multifaceted nature. By examining genetic, physiological, cognitive, social, and environmental factors, these studies underscore the importance of adopting multi‐factorial approaches to identify high‐risk individuals and inform targeted interventions. At the same time, the limitations of current methods, such as the reliance on self‐reports and the underrepresentation of diverse populations, remind us of the challenges that remain. Future research must aim to include more diverse samples, integrate multiple informants, and explore how the impact of combined factors varies over time. By addressing these gaps, we can move closer to developing comprehensive, equitable, and effective strategies to support adolescents at risk of internalizing disorders and improve mental health outcomes for this vulnerable population.

## AUTHOR CONTRIBUTIONS

**Pablo Vidal‐Ribas**: Conceptualization; writing—original draft.

## CONFLICT OF INTEREST STATEMENT

Pablo Vidal‐Ribas is a Joint Editor for *JCPP Advances* and is also supported by grant RYC2021‐033369‐I, funded by MCIN/AEI/10.13039/501100011033 and the European Union “NextGenerationEU/PRTR” and the CERCA program/Generalitat de Catalunya.

## FUNDING INFORMATION

MCIN/AEI/10.13039/501100011033; RYC2021‐033369‐I; European Union “NextGenerationEU/PRTR”; CERCA program/Generalitat de Catalunya

## ETHICAL CONSIDERATIONS

None.

## Data Availability

Data sharing not applicable to this article as no datasets were generated or analyzed.
